# The role of fibrinogen in predicting reinfection after DAIR for periprosthetic joint infections

**DOI:** 10.1186/s12891-021-04357-8

**Published:** 2021-05-24

**Authors:** Dacheng Zhao, Jinwen He, Xingwen Wang, Xiaobing Zhao, Yayi Xia, Bin Geng

**Affiliations:** Department of Orthopaedics, Lanzhou University Second Hospital, Orthopaedics Key Laboratory of Gansu Province, Orthopaedics Clinical Research Center of Gansu Province, No. 82 of Cuiying Men, Lanzhou, 730030 Gansu China

**Keywords:** Fibrinogen, Periprosthetic joint infection, Debridement, Implant retention, Reinfection

## Abstract

**Background:**

Fibrinogen (FIB) has been found to be a promising marker in diagnosing periprosthetic joint infection (PJI), however, the value of FIB in predicting reinfection of PJI is unknown. The purpose of this study was to evaluate the value of FIB in predicting reinfection after debridement, antibiotics, and implant retention (DAIR) for PJI.

**Methods:**

We retrospectively analyzed the clinical data of patients who were diagnosed with PJI and underwent DAIR from 2013 to 2019. The levels of the FIB, erythrocyte sedimentation rate (ESR), and C-reactive protein (CRP) were measured before DAIR. After DAIR, patients were followed and reinfections were identified. For both acute and chronic PJI, the predictive value of FIB was evaluated by calculating the sensitivity, specificity, and area under the curve (AUC) of the receiver operating characteristic curve (ROC), and was compared with traditional inflammatory markers including ESR and CRP.

**Results:**

The expression of FIB differed between patients reinfected and those not reinfected in both acute and chronic PJI (*p* < 0.05). In patients who underwent DAIR for acute PJI, the sensitivity and specificity of FIB were 81.82 and 83.33%, respectively, which were significantly higher than that of CRP (sensitivity, 72.73%; specificity, 50%; *p* < 0.05), while the specificity was higher than that of ESR (specificity, 41.67%; *p* < 0.05). In patients who underwent DAIR for chronic PJI, the sensitivity and specificity of FIB were 80.00 and 66.66%, respectively, which were significantly higher than that of CRP (sensitivity, 53.33%; specificity, 66.66%; *p* < 0.05) and ESR (sensitivity was 66.00%; specificity, 16.66%; *p* < 0.05). The ROC curves showed that FIB demonstrated the highest AUC among the biomarkers in both acute and chronic PJI.

**Conclusion:**

FIB is a promising indicator in predicting reinfection after DAIR for both acute and chronic PJI, and it seems to perform better than ESR and CRP.

**Supplementary Information:**

The online version contains supplementary material available at 10.1186/s12891-021-04357-8.

## Introduction

Periprosthetic joint infection (PJI) is a serious complication after primary hip and knee arthroplasty, and the primary reason for failure after surgery [1]. Treatment options for PJI include debridement, antibiotics, and implant retention (DAIR), 1-stage revision, 2-stage revision, joint fusion, and amputation [2–6]. The success rate of DAIR for PJI has been reported to be between 20 and 80% and related to obesity, comorbidities, operative time, perioperative period, microbial culture, and sensitivity to antibiotics [7–9].

Traditionally, diagnosis of infection after joint arthroplasty includes measuring serum levels of ESR and CRP [10–12], however, in the acute phase they can be influenced by the surgical intervention [13]. Furthermore, the formation of biofilms in the chronic stage may affect the utility of ESR and CRP to evaluate infection [14]. Therefore, new indicators are required for the assessment of acute and chronic PJI. FIB is a coagulation component that has been recently found to be an inflammatory marker useful in the diagnosis of PJI [15, 16]. Our previous study showed that FIB was not inferior to ESR and CRP in differentiating PJI and aseptic loosening, and it was useful in assessing infection outcomes after first-stage surgery [14]. Serum D-dimer, also been reported for use in PJI diagnosis [17], is included in the new validated diagnostic criteria of PJI [18]. A recent multicenter study revealed that fibrinogen (FIB) performed better than D-dimer in PJI diagnosis, with similar sensitivity and specificity compared with ESR and CRP [19]. To our knowledge, whether FIB can be used to predict reinfection after DAIR is unknown. The purpose of this study was to evaluate the value of FIB in predicting reinfection in patients who underwent DAIR for PJI.

## Materials and methods

### Patients

We retrospectively reviewed the database of arthroplasty surgeries in our center from 2013 to 2019. Patients who were treated with DAIR for PJI after primary hip and knee arthroplasty were included. The definition of acute and chronic PJI followed the MSIS standard [20], in which the acute phase was defined as within 90 days and chronic phase was defined as more than 90 days after index arthroplasty. Patients who were diagnosed with acute hematogenous infections were not specified in our study cohort. The exclusion criteria considered factors that may influence the levels of biomarkers, including debridement or revision surgery previously, use of anticoagulant or antibiotics 2 weeks before admission, venous thromboembolism (VTE), autoimmune disease, hematologic diseases, liver diseases, and malignancies. The study was approved by the Ethics Committee of our hospital (2019A-211).

### Preoperative evaluation

The 2018 criteria of the International Consensus Meeting (ICM) on musculoskeletal infection were used for the diagnosis of PJI [18]. After admission, the serum levels of ESR and CRP, and FIB were measured. An experiential antibiotic regimen of vancomycin (1 g, Q12h) and cefoperazone (3 g, Q8h) was used before the pathogen was identified. All patients underwent joint aspiration before DAIR with fluid sent for microbiologic examination. Blood and tissue samples were routinely cultured for 7 days with at least three specimens obtained. In culture-negative cases, tissue cultures were held for 14 days. Antibiotic regimens were tailored according to the identified pathogen and consultation from the pharmacy department. The interval between the index surgery and detection of infection and the identified pathogen were recorded. Methicillin-sensitive *Staphylococcus aureus* (MSSA) and methicillin-resistant *Staphylococcus aureus* (MRSA) were listed as subgroups of total *Staphylococcus aureus* isolates.

### DAIR and identification of reinfection

All patients underwent standard DAIR, tissues obtained intraoperatively were sent for pathological and microbiologic examination, and the results were confirmed by experienced experts. Antibiotics were given intravenously for 2 weeks and orally for 4 weeks, and the regimens were adjusted according to the symptoms and laboratory examination. All patients were followed up for at least 1 year. The criteria for reinfection after DAIR were (1) Any reoperation for infection after surgery. (2) Long-term use of inhibitory antibiotics (more than 3 months). (3) Recurrent infections that were caused by strains of the same organism. (4) PJI related deaths.

### Statistical analysis

The sample size required for this study was assessed by using PASS software (version 15) and the Power value asset 0.9. Continuous variables were expressed as mean and standard deviation, and assessed by using t-test. The rank-sum test was used when the homogeneity of variance of continuous variables was not equal. The predictive values of FIB, ESR, and CRP were evaluated by calculating the sensitivity, specificity, positive predictive value (PPV), and negative predictive value (NPV), and area under the curve (AUC) in the receiver operating characteristic (ROC) curve. AUC was defined as the area under the ROC curve and the coordinate axis, the discriminative power was defined as outstanding (0.90–1.00), good (0.80–0.89), general (0.70–0.79), the difference (0.60–0.69), or unqualified (0.50–0.59). The optimal cutoff was determined by calculating the Youden index. Comparison between variables was run by using the chi-square test. All statistical analyses were performed by using IBM SPSS statistical software (Version 25), MedCalc statistical software (version 19). The significance value was defined as 0.05.

## Results

From January 2013 to August 2019, 44 patients were included for analysis. Twenty-three patients were diagnosed with acute PJI (acute PJI group) and 21 patients were diagnosed with chronic PJI (chronic PJI group). There were no significant differences in demographic data including age, sex, body mass index (BMI), and involved joints between the two groups (*p* > 0.05, Tables 1 and 2). Patients were furtherly divided into subgroups according to whether they developed reinfection after DAIR. Eleven patients (47.8%) in the acute PJI group and 15 patients (71.4%) in the chronic PJI group were diagnosed with reinfection after DAIR. There was no significant difference in reinfection rate between the acute and chronic PJI groups (*p* = 0.136).

**Table 1:** Demographic information of patients with acute PJI treated with DAIR

Characteristic	Reinfection		*P* value
	Yes (*n* = 11)	No (*n* = 12)	
Age (y)	63.18 ± 8.62	63.75 ± 11.89	0.898
Gender			0.912
Male	2 (18.2%)	3 (25.0%)	
Female	9 (81.8%)	9 (75.0%)	
BMI (kg/m2)	25.32 ± 4.88	26.61 ± 4.06	0.499
Involved joint			0.296
Knee	6 (54.5%)	10 (83.3%)	
Hip	5 (45.5%)	2 (16.7%)	

All quantitative data were expressed as mean ± standard deviation

*PJI* Periprosthetic joint infection, *DAIR* Debridement, antibiotics, and implant retention, *BMI* Body mass index

**Table 2:** Demographic information with chronic PJI treated with DAIR

Characteristic	Reinfection		*P* value
	Yes (*n* = 15)	No (*n* = 6)	
Age (y)	64.27 ± 9.19	64.50 ± 3.51	0.934
Gender			0.944
Male	9 (18.2%)	3 50.0%)	
Female	6 (81.8%)	3 (50.0%)	
BMI (kg/m2)	24.85 ± 3.33	25.34 ± 2.39	0.749
Involved joint			0.831
Knee	9 (54.5%)	4 (66.7%)	
Hip	6 (45.5%)	2 (33.3%)	

All quantitative data were expressed as mean ± standard deviation

*PJI* Periprosthetic joint infection, *DAIR* Debridement, antibiotics, and implant retention, *BMI* body mass index

### Diagnose of PJI and pathogens

In the acute PJI group, the interval between index surgery and identification of PJI was 1.50 (1.10, 2.10) months in the reinfection patients and 1.25 (0.925, 1.95) months in the non-reinfection patients (*p* = 0.347). In the chronic PJI group, the interval between index surgery and identification of PJI was 11.50 (7.9–21.20) months in the reinfection patients and 12.50 (4.15–26.50) months in the non-reinfection patients (*p* = 0.622). The interval between index surgery and identification of PJI and the identified pathogens in each subgroup were shown in Tables 3 and 4.

**Table 3:** Biomarker levels before DAIR in patients with acute PJI

Items	Reinfection		*P* value
	Yes (*n* = 11)	No (*n* = 12)	
ESR (mm/h)	42.00 (38.00, 65.00)	39.00 (20.25, 49.50)	0.288
CRP (mg/L)	48.60 (27.54, 88.50)	61.44 (38.93, 103.12)	0.651
FIB (g/L)	4.03 (3.45, 4.61)	3.08 (2.92, 3.32)	< 0.05
Interval between index surgery and PJI (mon)	1.50 (1.10, 2.10)	1.25 (0.925, 1.95)	0.347
Culture			
*Staphylococcus epidermidis*	3 (27.3%)	2 (16.7%)	
Hemolytic streptococcus	2 (18.2%)	2 (16.7%)	
Total *Staphylococcus aureus* isolates	6 (54.5%)	8 (66.6%)	

The data were expressed as median (interquartile range)

*PJI* Periprosthetic joint infection, *DAIR* Debridement, antibiotics, and implant retention, *ESR* Erythrocyte sedimentation rate, *CRP* C-reactive protein, *FIB* Fibrinogen, *MSSA* Methicillin-sensitive Staphylococcus aureus

**Table 4:** Biomarker levels before DAIR in patients with chronic PJI

Items	Reinfection		*P* value
	Yes (*n* = 15)	No (*n* = 6)	
ESR (mm/h)	34.00 (17.00, 68.00)	35.50 (20.00, 72.50)	0.733
CRP (mg/L)	23.26 (5.62, 33.98)	13.06 (4.02, 32.22)	0.622
FIB (g/L)	4.28 (3.98, 5.12)	3.68 (3.20, 4.05)	< 0.05
Interval between index surgery and PJI (mon)	11.50 (7.9, 21.20)	12.50 (4.15, 26.50)	0.622
Culture			
Staphylococcus epidermidis	4 (26.7%)	1 (16.7%)	
Hemolytic streptococcus	2 (13.3%)	2 (33.3%)	
Negative	3 (20.0%)	1 (16.7%)	
Escherichia coli	2 (13.3%)	0	
Total Staphylococcus aureus isolates	4 (26.7%)	2 (33.3%)	
MSSA	2 (13.3%)	2 (33.3%)	
MRSA	2 (13.3%)	0	

The data were expressed as median (interquartile range)

*PJI* Periprosthetic joint infection, *DAIR* Debridement, antibiotics, and implant retention, *ESR* Erythrocyte sedimentation rate, *CRP* C-reactive protein, *FIB* Fibrinogen, *MSSA* Methicillin-sensitive Staphylococcus aureus, *MRSA* Methicillin-resistant Staphylococcus aureus

### Predictive value of biomarkers

In both acute and chronic PJI groups, there were no significant differences in the levels of ESR and CRP between the reinfection and no reinfection patients (*p* > 0.05, Tables 3 and 4), however, the levels of FIB in the reinfection group were significantly higher than that of the no reinfection group (*p* < 0.05, Tables 3 and 4).

**Fig. 1:**
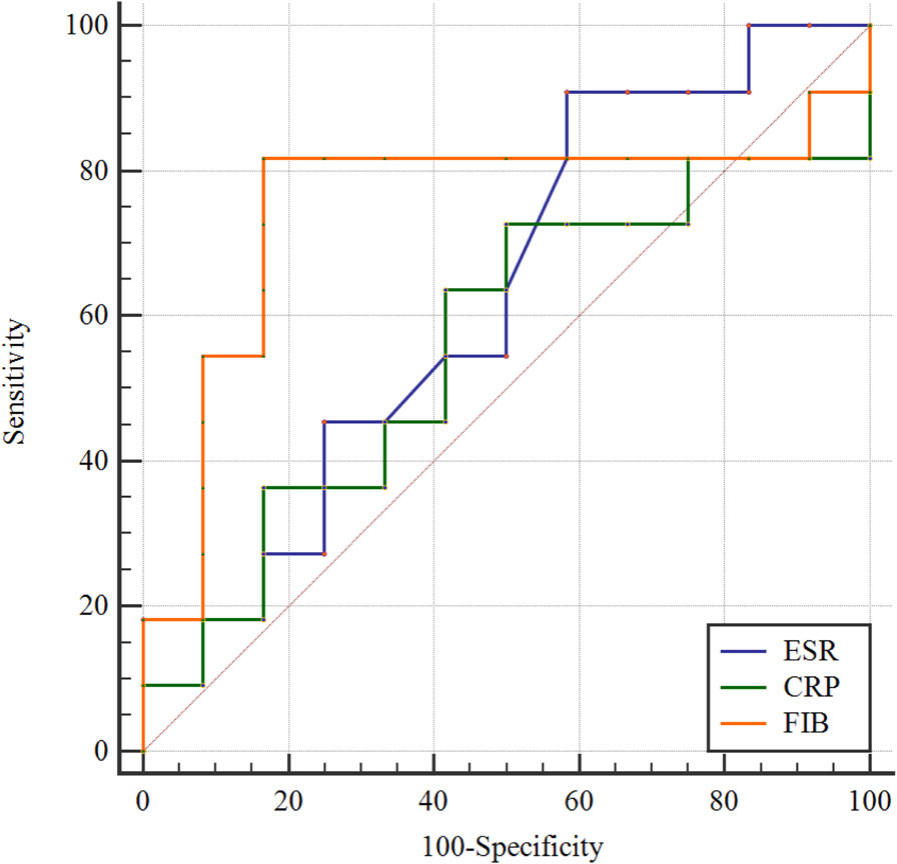
Receiver operating characteristic curve analysis for predicting reinfection after DAIR treatment for acute periprosthetic joint infection. DAIR debridement, antibiotics, and implant retention, ESR erythrocyte sedimentation rate, CRP C-reactive protein, FIB fibrinogen

In the acute PJI group, the ROC curves showed that FIB demonstrated the highest AUC of 0.75, with an optimal cutoff of 3.32. The sensitivity, specificity, PPV, NPV were 81.82, 83.33, 81.80, 83.33%, respectively. ESR showed an AUC of 0.63, the cutoff point was 22, the sensitivity, specificity, PPV, NPV were 90.91, 41.67, 58.80, 83.30%, respectively. CRP demonstrated an AUC of 0.56, with a cutoff point of 58.62, the sensitivity, specificity, PPV, NPV were 72.73, 50.00, 57.10, 66.70%, respectively (Fig. 1; Table 5).

**Table 5:** Diagnostic value of biomarkers for acute and chronic PJI treated with DAIR

Values of biomarkers	Acute PJI (*n* = 23), Reinfection (*n* = 11)	Chronic PJI (*n* = 21), Reinfection (*n* = 15)
ESR		
AUC	0.63	0.56
Optimal cutoff	22 mm/h	20 mm/h
Youden index	0.33	0.23
Sensitivity	90.91%	66.00%
Specificity	41.67%	16.66%
PPV	58.80%	66.66%
NPV	83.30%	16.66%
CRP		
AUC	0.56	0.58
Optimal cutoff	58.62 mg/L	16.50 mg/L
Youden index	0.23	0.37
Sensitivity	72.73%	53.33%
Specificity	50.00%	66.66%
PPV	57.10%	80.00%
NPV	66.70%	36.36%
FIB		
AUC	0.75	0.81
Optimal cutoff	3.32 g/L	3.94 g/L
Youden index	0.65	0.63
Sensitivity	81.82%	80.00%
Specificity	83.33%	66.66%
PPV	81.80%	85.71%
NPV	83.30%	57.14%

*ROC* Receiver operating characteristic, *ESR* Erythrocyte sedimentation rate, *CRP* C-reactive protein, *FIB* Fibrinogen, *AUC* Area under the curve, *PPV* Positive predictive value, *NPV* The negative predictive value

**Fig. 2:**
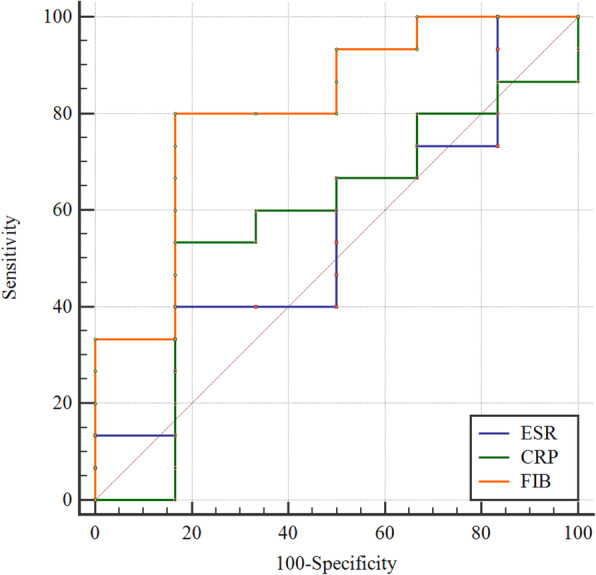
Receiver operating characteristic curve analysis for predicting reinfection after DAIR treatment for chronic periprosthetic joint infection. DAIR debridement, antibiotics, and implant retention, ESR erythrocyte sedimentation rate, CRP C-reactive protein, FIB fibrinogen

In the chronic PJI group, the ROC curves showed that FIB demonstrated the highest AUC of 0.81, with an optimal cutoff of 3.94. The sensitivity, specificity, PPV, NPV were 80.00, 66.66, 85.71, 57.14%, respectively. ESR showed an AUC of 0.56, the cutoff point was 20, the sensitivity, specificity, PPV, NPV were 66.00, 16.66, 66.66, 16.66%, respectively. CRP demonstrated an AUC of 0.58, with a cutoff point of 16.50, the sensitivity, specificity, PPV, NPV were 53.00, 66.66, 80.00,36.36%, respectively (Fig. 2; Table 5).

In summary, the AUC of FIB was highest among the three biomarkers. The sensitivity, specificity, PPV, and NPV of FIB were higher than that of ESR and CRP in the chronic PJI group. Similar results were observed in the acute PJI group except for sensitivity, FIB had a lower sensitivity than ESR.

## Discussion

In the present study, we found that FIB was a promising biomarker in predicting reinfection after DAIR for both acute and chronic PJI, and it seemed to perform better than ESR and CRP. We reviewed the literature and confirmed that this is the first study assessing the predictive value of FIB after DAIR for acute and chronic PJI.

Management of PJI is challenging. DAIR is recommended for acute PJI by the International Consensus [21], as it avoids reimplanting the prosthesis and can decrease the risk of complications. DAIR for chronic PJI is also reported [22]. Since the failure of DAIR is common in clinical practice, especially for chronic PJIs, the identification of those at increased risk for reinfection after DAIR is crucial.

The detection of reinfection after DAIR has been previously reported. Maier et al. [23] found that ESR had a sensitivity of 58%, and a specificity of 66% for reinfection after DAIR performed for acute postoperative infection; while CRP showed a sensitivity of 42%, and specificity of 56%. For chronic infection, ESR showed a sensitivity of 67%, and a specificity of 47%, while CRP showed a sensitivity of 50% and a specificity of 26%. Kuiper et al. [24] found that the failure rate of DAIR increased when ESR was higher than 60 mm/h. Similarly, a multicenter retrospective study showed that a high CRP level was associated with failure after DAIR [8]. Though ESR and CRP are traditional inflammatory indicators that are widely used to evaluate postoperative infections [12, 25, 26], they can be influenced by surgical intervention. Previous studies found that the value of a single biomarker is limited in diagnosing PJI. Researchers attempted to combine single biomarkers into a multi-biomarker model to improve diagnostic properties, and found that CRP and FIB performed best amongst all biomarkers. However, the construction of a multi-biomarker model was not feasible [27].

The present study demonstrated 47.8 and 71.43% reinfection rates after DAIR for acute and chronic PJI respectively, which is similar to previous studies [28]. With regard to ESR and CRP in predicting failure, our results of specificity in the acute group and sensitivity in the chronic group were similar to that of Maier et al. [23], while sensitivity in the acute group and specificity in the chronic group were higher than their findings. Additionally, we showed a higher AUC for ESR and CRP, in both the acute and chronic PJI patients.

We found that in comparison to CRP, FIB had higher sensitivity and specificity in both acute and chronic reinfections. In comparison to ESR, FIB had higher specificity in acute reinfection and higher sensitivity and specificity in chronic reinfection. Since data of FIB in predicting reinfection after DAIR is lacking, the comparison of FIB with other studies was not available. However, our results revealed that FIB had a better discriminative power in predicting reinfection, indicating that FIB appears to be a screening indicator of reinfection for patients undergoing DAIR for both acute and chronic PJI. The ideal biomarker for any condition should be reliable and reproducible, sensitive and specific, and provide risk stratification. Since FIB is a component of the coagulation test and is originally performed after admission, it is convenient and cost-effective compared to other inflammatory biomarkers.

The application of FIB does have several limitations, including that levels could be influenced by anticoagulant use, and that patients with thromboembolic disease, autoimmune disease, liver disease, among other conditions, were excluded in this study. As a result, how applicable FIB would be in the real world remains to be seen. Additionally, the study design was retrospective with a small sample size and interpretation may be influenced by selection bias and the composition of the study cohort. Furthermore, outcomes in PJI could be influenced by drug sensitivity, antibiotic regimen, comorbidities, and duration of the infection [29, 30]. Furthermore, it was difficult to design a well-matched comparison group. Large, randomized controlled prospective studies are necessary to further confirm our findings.

## Conclusion

FIB is a promising indicator in predicting reinfection after DAIR for both acute and chronic PJI, and it seems to perform better than ESR and CRP.

## Supplementary Information

**Additional file 1.**

## Data Availability

All data generated or analyzed during this study are included in this published article [and its supplementary information files].

## Abbreviations

CRP: C-reactive protein
ESR: Erythrocyte sedimentation rate
FIB: Fibrinogen
MSIS: Musculoskeletal Infection Society
PJI: Periprosthetic joint infection
DAIR: Debridement, antibiotics, and implant retention
WBC: White blood cell
ROC: Receiver operating Characteristic curve
AUC: Area under the curve
PPV: Positive predictive value
NPV: Negative predictive value
VTE: Venous thromboembolism
